# Evolutionary Sequence Modeling for Discovery of Peptide Hormones

**DOI:** 10.1371/journal.pcbi.1000258

**Published:** 2009-01-09

**Authors:** Kemal Sonmez, Naunihal T. Zaveri, Ilan A. Kerman, Sharon Burke, Charles R. Neal, Xinmin Xie, Stanley J. Watson, Lawrence Toll

**Affiliations:** 1 SRI International, Menlo Park, California, United States of America; 2 Molecular and Behavioral Neuroscience Institute, University of Michigan, Ann Arbor, Michigan, United States of America; 3 AfaSci, Burlingame, California, United States of America; UT Southwestern Medical Center, United States of America

## Abstract

There are currently a large number of “orphan” G-protein-coupled receptors (GPCRs) whose endogenous ligands (peptide hormones) are unknown. Identification of these peptide hormones is a difficult and important problem. We describe a computational framework that models *spatial* structure along the genomic sequence simultaneously with the *temporal* evolutionary path structure across species and show how such models can be used to discover new functional molecules, in particular peptide hormones, via cross-genomic sequence comparisons. The computational framework incorporates *a priori* high-level knowledge of structural and evolutionary constraints into a hierarchical grammar of evolutionary probabilistic models. This computational method was used for identifying novel prohormones and the processed peptide sites by producing sequence alignments across many species at the functional-element level. Experimental results with an initial implementation of the algorithm were used to identify potential prohormones by comparing the human and non-human proteins in the Swiss-Prot database of known annotated proteins. In this proof of concept, we identified 45 out of 54 prohormones with only 44 false positives. The comparison of known and hypothetical human and mouse proteins resulted in the identification of a novel putative prohormone with at least four potential neuropeptides. Finally, in order to validate the computational methodology, we present the basic molecular biological characterization of the novel putative peptide hormone, including its identification and regional localization in the brain. This species comparison, HMM-based computational approach succeeded in identifying a previously undiscovered neuropeptide from whole genome protein sequences. This novel putative peptide hormone is found in discreet brain regions as well as other organs. The success of this approach will have a great impact on our understanding of GPCRs and associated pathways and help to identify new targets for drug development.

## Author Summary

Peptide hormones, or neuropeptides, are made up of a string of amino acids ranging from approximately 3 to 50 residues. These peptides are processed from a larger protein called a prohormone and activate a class of proteins called G-protein-coupled receptors (GPCRs). Neuropeptides signal neurons and other cells leading to changes in cellular biochemistry and potentially gene expression. There are a number of “orphan” GPCRs, i.e., receptors that have been discovered either by genomic sequence or by cloning, in which its respective peptide hormone is unknown. We have devised a computational method that models patterns in protein sequence simultaneously with evolutionary differences across species in order to identify previously unknown peptide hormones. We have used this computational methodology to identify a previously unknown putative prohormone that contains up to four potential neuropeptides, and we have characterized this prohormone with respect to location in rat brain and various human tissues. This computational technique will be useful for the identification of additional neuropeptides and help to characterize orphan GPCRs. Because roughly half of all pharmaceuticals act through activation or inhibition of GPCRs, this technique should lead to the identification of additional pharmaceutical targets and ultimately clinically used drugs.

## Introduction

G protein coupled receptors (GPCRs) probably represent the largest gene family, making up 3% of the mammalian genome [1]. These proteins are made up of several subfamilies, including Class A rhodopsin-like, Class B secretin-like, Class C metabotropic glutamate/pheromone-like, and other nonmammalian receptors. Within each class, there is a very large number of smaller subclassifications, such as a family of receptors for peptide hormones within rhodopsin-like receptors. There are approximately 1,000 GPCRs, the vast majority of which are olfactory receptors, with more than 650 GPCRs in the rhodopsin family alone [2]. A large number of these receptors have been identified only by computational methods, while others have been cloned and transfected into cells; however, the cognate neurotransmitter and the receptor functions for many GPCRs are currently unknown. Any receptor for which the native neurotransmitter is unknown is considered an orphan receptor. Of all the orphan receptors that remain, some percentage represents receptors for peptide hormones.

This large family of proteins is important not only from a basic science perspective, but because of their extracellular sites of action and importance as first messengers for cellular signaling, GPCRs have become a primary target for drug development. In fact, over 30% of all pharmaceuticals act either as agonists or antagonists of GPCRs [3]. Many pharmaceutical companies are identifying, cloning, and patenting new orphan GPCRs, with the hope that orphan receptors will ultimately lead to new drug development and new pharmaceutical agents.

Although the identification of putative GPCRs can be accomplished relatively easily, the discovery of the endogenous ligands that activate these receptors is far more difficult. These ligands can exist as small molecules, lipids, peptides, or proteins [4],[5]. Many, such as ATP, may have important functions other than activating a GPCR. Even within a class of hormones, there are seldom obvious clues that identify a new candidate. This is particularly true within the family of peptide hormones, as they are processed from a larger species known as preprohormones [6].

Peptide hormones, or neuropeptides, are a string of amino acids ranging from approximately 3 to 50 residues. They are found within a larger protein (a preprohormone), and the production of the actual hormone usually follows specific rules. Preprohormones are secreted proteins, and each has a signal sequence that is necessary for the transport of the protein out of the Golgi complex into a secretory vesicle for processing and secretion where the signal sequence is removed, revealing the prohormone [7]. In general, hormones are surrounded by a pair of basic residues, *i.e.* Arg-Arg, Arg-Lys, Lys-Arg, or Lys-Lys, which are found directly adjacent to the putative hormone. These double basic residues act as recognition sites for processing enzymes, usually serine proteases that cleave the prohormone to liberate the active peptide [7],[8]. In many cases, there is more than a single active peptide within one precursor protein [6].

Even with these common features, the identification of a peptide hormone from a DNA or protein sequence is very difficult. Even though all of the GPCRs are obviously related based upon DNA or protein sequence, the neuropeptides that bind to the receptors are only obviously related within discrete families of prohormones. For instance, the family of opioid-like peptides has four members. These prohormones, proopiomelanocortin (POMC), proenkephalin, prodynorphin, and pronociceptin (proN/OFQ), share similar genomic structures and a very slight similarity of protein sequence, most notably the Y(F)GGF of enkephalin, β-endorphin, dynorphin, and N/OFQ [9],[10]. However, if one were to conduct a BLAST search in Genbank for DNA sequences similar to proenkephalin, one would not find any other neuropeptide. Simple search strategies within Genbank are not adequate for identifying novel neuropeptides, especially those not belonging to known neuropepeptide families.

There is an additional feature of neuropeptides that may more clearly differentiate them from other types of molecules. Neuropeptides are usually well conserved among various species (rat, mouse, human), while the intervening sequences, presumably because they are simply discarded, are not well conserved [11]. Here we describe a novel Hidden Markov Model (HMM)-based computational framework, the Match Profile HMM (MPHMM) method for neuropeptide identification based upon an approach that models *spatial* structure along the genomic sequence simultaneously with the *temporal* evolutionary path structure across species, and show how such models can be used to discover new functional molecules via cross-genomic sequence comparisons. This computational tool was used to identify a novel prohormone, NPQ, containing up to four potential neuropeptides [12]

## Results

### Computational Modeling of Preprohormone Evolution by a Hierarchical Grammar of Evolutionary Probabilistic Models

#### Hierarchical grammars of MPHMM modules

**Figure 1:**
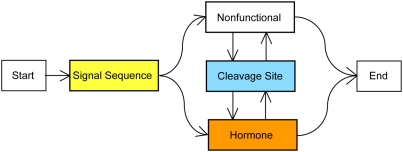
Prohormone hierarchical grammar of evolutionary MPHMM modules.

Hierarchical grammars of evolutionary HMMs, such as phylo-HMMs or MPHMMs are probabilistic models that take into account the way substitutions take place in the evolutionary path at specific sites along the genome, and the specific patterns of change from one site to the next. Figure 1 shows a hierarchical grammar of evolutionary HMM modules for a preprohormone. At the functional-level hierarchy, the model is specified in terms of its functional elements, which are signal sequences, cleavage sites, and preserved and diverged regions. The underlying evolutionary HMM modules carry out the local multiple alignments with respect to the phylogenetic relationship warranted by the context. This kind of hierarchical alignment is significantly more informative than a conventional multiple sequence alignment in that it provides a segmentation that has to satisfy higher-level constraints. For example, for the peptide hormone problem, the most important feature of a cross-genome alignment turns out to be the difference between the substitution rates of the functional and the nonfunctional subsequences around (predominantly double basic residue) splicing sites.

There are several formalisms for describing probabilistic evolutionary algorithms in the literature. We follow the exposition [13] used for the phylo-HMMs. Let us define the computational structure of a hierarchical grammar of functional-evolutionary model modules (MPHMMs or phylo-HMMs) by the four-tuple 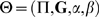, where 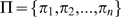 is a set of functional component states (for functions such as a signal sequence, a splicing site, or a peptide) with the set of associated functional element models, 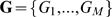, with the model 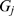 accounting for the part of the sequence alignment at the component state 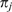. 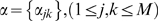, and 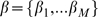 are the matrix of component state transition probabilities and the vector of initial probabilities, respectively. In this formulation, for the sake of descriptive efficiency, we are describing the basic two-level hierarchy of models, which can, in our implementation, entail more levels. In the lower level of the hierarchy, each component model is a vector output HMM with an alphabet consisting of the four-tuple, 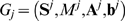, where 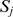 is a set of states associated with the functional component module. For example, a simple double basic residue cleavage site HMM would have two states that emanate multiple alignments of Arg and Lys residues. The set of associated functional element models, 
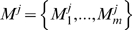
 account for the amino acid sequence with 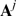 and 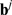 as the matrix of lower level state transition probabilities and the vector of initial probabilities, respectively. This structure also supports hierarchical grammars of phylo-HMMs [13]. In that case, 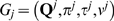, where 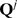 is the substitution matrix defined with respect to the alphabet of amino acids, 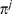 is a vector of equilibrium frequencies, 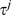 is the binary phylogenetic tree with the set of branch lengths 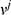. For phylo-HMMs, Felsenstein's “pruning” algorithm [14] is used for the phylogenetic model optimization rather than Viterbi for our model.

**Figure 2:**
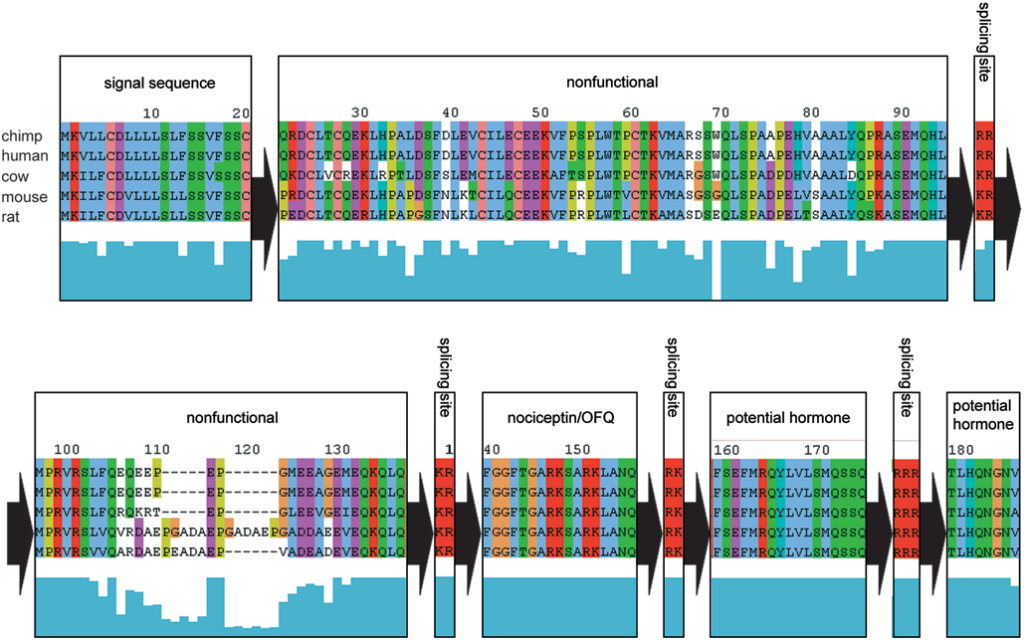
Hierarchical functional-element multiple alignment of Pronociceptin across human, chimpanzee, mouse, rat, and cow.

In this two-level hierarchical approach, there are two types of alignments, (i) functional alignments at the high level, 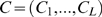, and (ii) state module alignments at the lower level, 
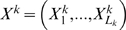
, 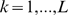. To illustrate this point, Figure 2 shows a hierarchical alignment for prepronociceptin from five species (human, chimp, mouse, rat, cow), where the boxes depict the functional element sequence. The resulting sequence alignments within functional elements are also shown.

A path through the functional element sequence is a sequence of states 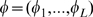, and a path through a component module is a sequence of states 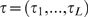. Given the above setting, we compute the joint probability of a functional level path and alignment, which is given by
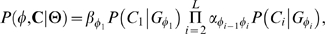
where, in turn, each of the functional module state alignments is given by
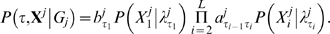
The likelihood of the model 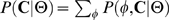 is found by summing over all possible paths, and the maximum likelihood path is the path that maximizes 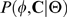. The computation of these quantities and the state posterior probabilities are facilitated by the Markovian structure that allows standard dynamic programming based solutions through the use of Viterbi and forward-backward algorithms.

#### Component MPHMM modules

MPHMMs account for the structural constraints of a preprohormone sequence by modeling separate modules in a combined manner by a modular profile HMM for each genome. The two modular HMMs for the two genomes are then coupled by several pairwise HMMs on a module-by-module basis across the two genomes in order to model differential evolutionary rates of functional and nonfunctional sequences. We name the overall framework Hierarchical Grammar of Hmms of Evolutionary Regions (HIGHER).

**Figure 3:**
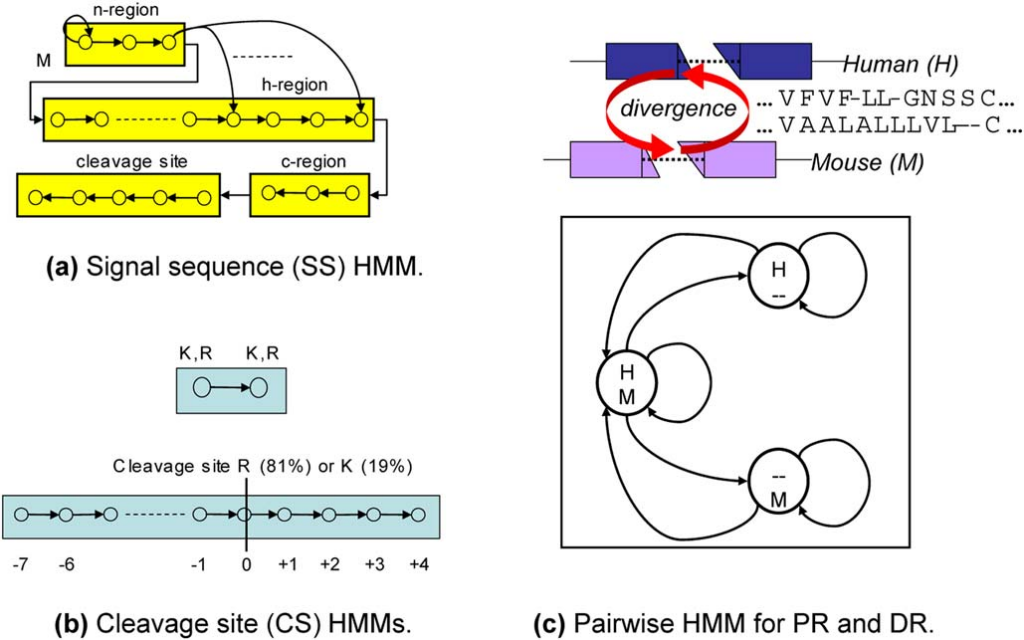
HIGHER MPHMM modules. (a) Signal sequence, (b) cleavage site, and (c) peptide/divergent region modules.

The structural topology of the modules comprises a signal sequence module, nonfunctional preprohormone module, splicing site module, and the functional hormone module in various possible combinations. Specifically, the signal sequence HMM module is shown in Figure 3a. This is essentially a topology similar to the HMM topology used by Nielsen et al. [15],[16], which models a general signal sequence with the requisite sites and results in a similar detection performance as that of SignalP [17].

There are two possible topologies for splicing site modules, as shown in Figure 3b, for two adjacent basic residues, and for a single basic residue [18]. Two consecutive basic residues is the simplest splicing site model, consisting strictly of two K or R residues in sequence, and is sufficient for the majority of known peptides. A single basic residue splice site occurs for an important number of peptides, though, and the model shown in Figure 3b, in which a single residue of K or R occurs in a context with a specific residue profile, can be trained with synthetic data generated based on sequences published by Devi [18].

The relative homology between hypothesized peptide hormone and divergent sections is modeled through the use of pairwise-HMMs, or their straightforward generalizations to multiple alignments of 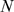 sequences in which all 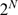 subsets occupy a separate state. Figure 3c depicts the structure of the pairwise-HMM for aligning two sequences. The relative difference between the homologies of the hypothesized and divergent regions produces the most informative feature from the alignment of multiple sequences to determine if the aligned sequences constitute a preprohormone by satisfying both the structural and the evolutionary constraints.

#### Computational processing steps

Two-dimensional statistical models that make extensive use of graphs, such as phylo-HMMs and HIGHER, are usually quite costly to compute. Estimation of models based on alignments of a multitude of genomes (more than five for example) requires considerable resources in terms of both CPU power and data. This fact limits their applicability as general filters or data mining tools that operate on large repositories of sequences for discovery. In our processing of all the protein sequences that were available to us, we had to address this difficulty, for which we used the hierarchical structure to our advantage by first forming initial raw alignments based on parsing of sequences with our functional element grammar, and aligning based on functional element identity alone. Then, the resulting sequences were realigned by the HIGHER model in order to obtain the fine alignment as well as the discriminatory features. More specifically, processing of the sequences followed three main steps: two multiple alignment steps; (i) raw multiple alignment via functional element detection, and (ii) fine multiple alignment via fitting of the MPHMM, and (iii) a final discrimination step where a score is generated from the multiple alignment. After the sequences were processed and scored, alignments were generated, and the biologists were provided with the list of hits in a graphical user interface. This interface was used to browse the list of hits with a more discriminatory viewing tool that includes constraints to filter the list of hits, e.g. according to region, lengths or maximum divergence.

### Summary of processing steps

**Figure 4:**
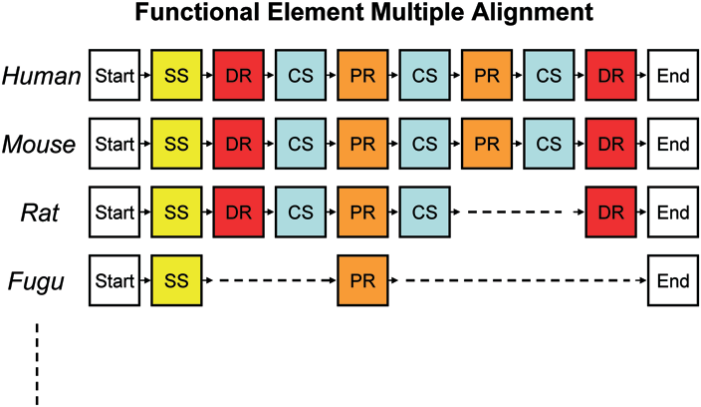
Multiple alignment of functional element sequences across genomes.

1. **Functional element transcription** of protein sequences from several genomes using the detector HMM modules and the preprohormone grammar. See Table 1 for modules and their abbreviations.
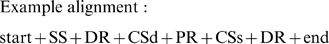

2. **Multiple functional element alignment** of protein sequences (Figure 4)
3. **Fit HIGHER model** to the multiple sequence alignment
4. **Browse the matches via the user interface** *SequenceMatcher* in the feature space to evaluate the hits (see http://www.cslu.ogi.edu/people/sonmezk/hormone).

**Table 1:** Modules and their abbreviations.

Functional Element	Symbol
Signal sequence	SS
Cleavage site (double basic)	CSd
Cleavage site (single basic)	CSs
Peptide hormone region	PR
Divergent region	DR

### Availability of Human-Mouse Search Results, SequenceMatcher, and the HIGHER Tools

The extended list of matches, the GUI SequenceMatcher, and the HIGHER tools will be made are available at http://www.cslu.ogi.edu/people/sonmezk/hormone. Initially, we will enable the visualization of our ENSEMBL and CELERA runs via the GUI. The next version will allow evolutionary HMM searches specified by the user. The HIGHER codebase will also be made available at the website once it is ready for release.

#### Search of SwissProt Database

As a proof of principle, we present results on SwissProt 41, a database containing a large number of known hormones. Because the functions of all of the proteins in SwissProt are known, this search does not produce novel peptide hormones, but it produces a detection metric for the performance of the search paradigm. Note that the structural profile HMMs for the signal sequence and the splicing sites have not been trained with these proteins, and in HIGHER we do not train sequence structure models for hormones, so our SwissProt set constitutes an independent test set. For one specific threshold, we were able to identify 45 out of 54 preprohormones known to be in SwissProt with 44 false alarms (Table 2). In terms of detection performance, this corresponds to a point on the receiver operating characteristic (ROC) curve with sensitivity of 83%, and specificity of more than 99.9% (44 false hits on a SwissProt set with 122,564 proteins).

**Table 2:** Matches Found in Swiss-Prot Database.

Hormones	Sequence Matching “Hits”	Hormones	Sequence Matching “Hits”
ACTH	x	MCH (melanin concentrating hormone)	
ADM (adrenalmedulin)	x	Motilin	x
Agouti-related peptides	x	MSH (melanocyte stimulating hormone)	x
Amylin	x	Neuromedin U	x
ANP (atrial natruretic peptide)		Neurotensin	x
Apelin		Neurturin	x
Calcitonin	x	Nociceptin	x
CART (cocaine and amphetamine regulated transcript)	x	NPY (neuropeptide Y)	x
CCK (cholecystokinin)	x	Orexins	x
CGRP (calcitonin gene related protein)	x	Oxytocin	x
CNP (C-type natriuretic factor)	x	PACAP (pituitary adenylate cyclase activating polypeptide)	x
Cortistatin		PPY (pancreatic hormone)	x
CRF (corticotropin releasing factor)	x	PHI (same precursor with VIP)	x
Dynorphin	x	PrRP (prolactin-releasing peptide)	
β-Endorphin	x	PTH (parathyroid hormone)	x
Endothelin 1	x	PTH-RP (parathyroid releasing hormone)	
Endothelin 2	x	PYY (peptide YY)	x
Endothelin 3	x	Secretin	x
Enkephalin	x	Somatostatin	
Galanin	x	Substance K ( = neurokinin A)	
Gastrin	x	Substance P	x
Glucagon	x	TEGT (testis enhanced gene transcript)	x
GRF (growth hormone releasing factor)	x	TRH (thyroid releasing hormone)	x
GRP (gastrin releasing peptide)		Vasopressin	x
Guanylin		VIP (vasoactive intestinal peptide)	x
LHRH1 (luetinizing hormone releasing hormone)	x	PSP94 (prostate secretory protein)	x

**False Positives**

Other signaling molecules: FGF-3,5,7,10,17,18; GDNF; CD8,28; PDGF-2; TGF; VEGF (vascular endothelial growth factor); HBNF-1; MIP; NGF (nerve growth factor); Cytokine A21, IFN-α (interferon alpha); IGF binding protein 1B,2,3; IL7 (interleukin 7).

Other: MAGF (microfibril associated protein), MINK (K-channel), K-channel related peptide, L-type Ca^2+^ channel, gamma subunit, myelin Po protein, Dif-2, Eosinophil, Syntaxin 1B (vesicle docking), Syntaxin 2, TMP21 (vesicle trafficking protein), Coagulation factor III, PGD2 synthase, syndecans, FKBP12 (FK506 binding protein), Folate receptor, ERp29, COMT, Connexin 32, Cytostatin.

#### Search of the Celera Database

We then collected the full list of known and putative proteins from mouse and human genomes using the Celera Discovery System (CDS) database. These two sets of proteins were matched using HIGHER and the resulting output examined for known and potentially novel peptide hormones. Each potential match was examined using the CDS that lists families to which these unknown proteins might belong. BLAST searches were also conducted on both the predicted protein and the mouse and human gene, using both CDS and Genbank. A gene family was evident for many of the potential matches, suggesting that these proteins did not represent novel neuropeptides. For a smaller number of matches, the function of the protein was unknown. We consider these to be potential novel preprohormones.

**Figure 5:**
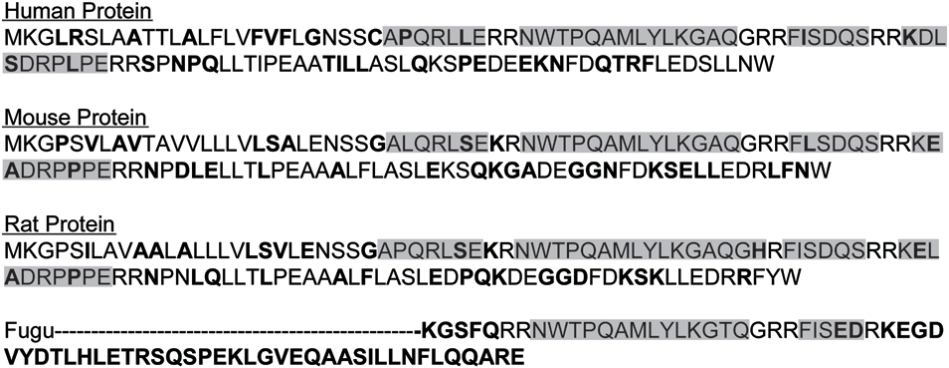
Amino acid sequence of preproNPQ. Sequences shown were obtained from GenBank. The human and rat sequences were verified by nucleotide sequencing as described in Materials and Methods. Putative neuropeptides highlighted. They begin at the end of the signal sequence and end at the fourth set of basic residues. Residues that are not conserved between human and other species are in bold.

One novel protein identified is the perfect example of our hypothetic neuropeptide model, shown in Figure 5. Between double basic residues, the homology is high. Outside these residues the conservation is quite low. The protein sequence of the human and rat were predicted from gene finding programs. These proteins have no apparent homology to any other proteins, and no known biological function. Of the four potential neuropeptides (highlighted in yellow, beginning at the end of the signal sequence and ending at the fourth set of basic residues), the most likely candidate would be the NPQ peptide NWTPQAMLYLKGAQ-NH_2_, although we should emphasize that one or more of the others (APQRLLE, FISDQS, and KDLSDRPLPE) are also likely to have biological activity. This amidated 14 amino acid peptide (we expect the G before the RR to be a substrate for the amidating enzyme peptidylglycine a-amidating monooxygenase, PAM [19]) is fully conserved among human and mouse. A further search of homologies for this protein found strong conservation for the amidated 14 amino acid peptide as far back as fugu. The fact that this portion of the protein is so highly conserved, including amidation and processing sites, strongly suggests the importance of this peptide sequence.

One interesting mutation in the rat gene is not found in the human, mouse, bovine, porcine or fugu protein. The rat protein has a mutation in the GRR at the C-terminal portion of the NPQ peptide. A single nucleotide change produces the sequence Gly-His-Arg. This complicates the processing of the rat gene product. It is possible that an endopeptidase may function at a His-Arg bond, and if so, it would become a substrate for carboxypeptidase E (CPE) [20], and the processed peptide would end Gln-Gly-His, without the amidation of the more abundant analog.

**Figure 6:**
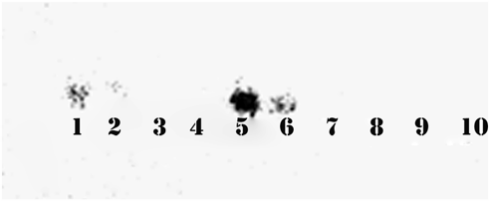
Northern Blot Analysis of preproNPQ mRNA. Ambion's First Choice Human Blot was prehybridized and probed with human NPQ cDNA prepared from the human DNA clone in pOTB7 vector from ATCC (Cat # 6710068, Manassas, VA). This clone contained the putative sequence for human NPQ. Random-prime labeling was performed using ^32^P-dCTP and Klenow DNA polymerase was conducted as described in Materials and Methods. 1. Brain, 2. Placenta, 3. Skeletal muscle, 4. Heart, 5. Kidney, 6. Pancreas, 7. Liver, 8. Lung, 9. Spleen, 10. Colon.

ESTs for preproNPQ have very recently appeared in GenBank indicating that the human protein can be found in brain, ovary, kidney and lung cancer cells. Our preliminary investigation of preproNPQ using RT-PCR shows the presence of its transcripts in human, mouse, and rat brains (Data not shown). We have cloned and sequenced the human, mouse, and rat cDNAs, and have verified the single nucleotide change that leads to the GHR sequence in the rat preproNPQ gene. Northern analysis using a human tissue blot (Clontech) showed the presence of preproNPQ mRNA in brain and pancreas, but most prominently in the kidney (Figure 6). Therefore, NPQ may be one of many peptides (such as vasopressin) found in both brain and kidney.

**Figure 7:**
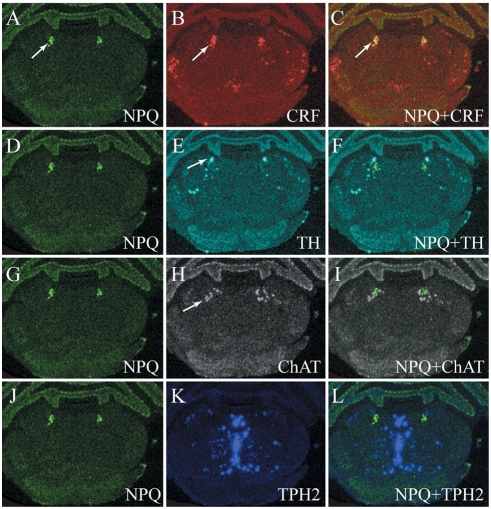
In situ hybridization of preproNPQ mRNA. Expression of preproNPQ mRNA in the rat brain at the level of Barrington's nucleus and locus coeruleus. *In situ* hybridizations (ISHs) for preproNeuropeptide Q (NPQ; A, D, G, and J), corticopin-releasing factor (CRF; B), tyrosine hydroxylase (TH; E), choline acetyltransferase (ChAT; H), and tryptophan hydroxylase 2 (TPH2; K) were carried out on adjacent 10 µm-thick sections of the rat brain. ISH autoradiograms were digitized; images were then inverted and pseudocolored according to the following scheme: NPQ – green, CRF – red, TH – cyan, ChAT – white, and TPH2 – blue. To determine whether NPQ signal overlapped with any of the other signals, the sections were aligned and overlaid with each other (C, F, I, L). Arrow in panel A indicates location of NPQ mRNA, while arrow in panel B indicates location of CRF mRNA; note the mixing of red and green (to yield yellow) in panel C (arrow) that suggests co-localization of NPQ and CRF. Arrow in panel E indicates locus coeruleus and its TH-positive neurons. Panel F shows that TH and NPQ signals are spatially very close without overlap. Arrow in panel H indicates the cholinergic laterodorsal tegmental nucleus, while panel I illustrates close spatial relationship between ChAT and NPQ mRNAs. At this level of the neuraxis there is little overlap between TPH2 mRNA (blue signal in panel K, which represents serotonergic neurons) and NPQ (L).

We have also conducted studies to determine regional localization in brain by *in situ* hybridization (Figure 7). An initial mapping study of preproNPQ mRNA demonstrated that its expression in the brain is restricted to the mesopontine tegmentum. At its caudal extent preproNPQ mRNA is confined to the Barrington's nucleus, which can be identified by its expression of corticotrophin releasing factor (CRF) mRNA (Figure 7B, arrow). As illustrated in Figure 7A–C, regional distribution of preproNPQ mRNA overlaps closely with that of CRF, suggesting possible cellular co-localization of these two mRNAs. In contrast, preproNPQ signal is distinct from that of tyrosine hydroxylase (TH) (Figure 7D–F), which is selectively expressed in locus coeruleus (Figure 7E, arrow). PreproNPQ mRNA is also closely related to, but does not overlap with, choline acetyltransferase (ChAT) (Figure 7G–I), which is expressed within the laterodorsal tegmental nucleus (Figure 7H, arrow). At this level of the neuraxis preproNPQ mRNA is located quite a bit lateral to the majority of the midline serotonergic neurons, as determined by examination of mRNA distribution of the synthetic enzyme tryptophan hydroxylase 2 (TPH2) (Figure 7J–L), though there is some overlap with the laterally displaced TPH2-positive neurons (Figure 7L).

**Figure 8:**
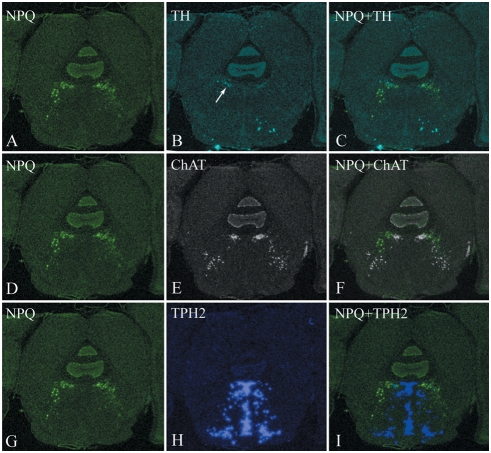
In situ hybridization of preproNPQ mRNA. Expression of preproNPQ mRNA at the level of the caudal ventrolateral periaqueductal gray (PAG). ISH autoradiograms were digitized and pseudocolored according to the same scheme as in Figure 7. NPQ signal was visible in the ventrolateral quadrant of the PAG as well as within the underlying reticular formation (A, D, G). ISHs for TH (B), ChAT (E) and TPH2 (H) were carried out on adjacent sections. Arrow in panel B indicates location of dopaminergic TH-positive neurons of the ventrolateral PAG that appear to overlap with a subset of NPQ mRNA (C). There is also close spatial relationship between NPQ and ChAT (F) and NPQ and TPH2 (I). Abbreviations are the same as in Figure 7.

At the level of the caudal periaqueductal gray (PAG), preproNPQ mRNA expression is restricted to the ventrolateral quadrant of this structure (Figure 8) with some scattered signal in the underlying reticular formation. Caudal ventrolateral PAG is a heterogeneous brain region that contains dopaminergic, cholinergic and serotonergic neurons. To determine whether preproNPQ mRNA signal overlaps with any of these populations, in situ hybridization (ISHs) for TH, ChAT and TPH2 were carried out. ISH for TH showed a weak but specific signal within the ventrolateral PAG (Figure 8B, arrow) that overlapped with preproNPQ signal (Figure 8A–C). ChAT mRNA was closely related to the preproNPQ signal but did not appear to overlap with it (Figure 8D–F). Likewise, laterally-displaced TPH2 mRNA was in close proximity to preproNPQ mRNA (Figure 8G–I).

## Discussion

Because devising computer-generated methods of identifying peptide hormones has been difficult, biochemical methods have been the most relied upon. Although they are time consuming and expensive, these methods work if one has some preliminary information or basic assumptions. Substance P was discovered based upon the physiological actions of brain extracts [21], while the peptide hormones met- and leu-enkephalin were discovered based upon a preexisting receptor [22]. Hughes and Kosterlitz used a smooth muscle bioassay for opiate receptors to isolate two peptides from bovine brain that were subsequently found to bind to the opiate receptors [22]. It was only several years later that these two peptides were found to be generated from a single prohormone [23]. Mutt and colleagues used a chemical assay to identify carboxy terminal amidated peptides, and in this way discovered neuropeptide Y (NPY) and peptide YY [24]. The purification and sequencing of N/OFQ (formerly nociceptin/orphanin FQ) was possible because of the availability of CHO cells transfected with NOP (N/OFQ peptide) receptors (formerly called ORL1) and the knowledge that the endogenous ligand would inhibit cAMP accumulation, as do the endogenous ligands for μ, δ, and κ opioid receptors, the other receptors in that family [25],[26]. Other examples of “reverse pharmacology” have followed, i.e. [27], and each has led to great strides in the understanding of human physiology.

Even though neuropeptides have very few apparent similarities as a class, computational tools can be used to characterize and even potentially identify new members of this class of signaling molecules. Bakalkin and colleagues have examined the bioinformatics of neuropeptides [28],[29]; they have computed the amino acid composition and relative amino acid arrangements in the neuropeptide portion and compared them to the intervening portions of a prohormone. Using this statistical method, they have found an increased content of certain residues, as well as an increased occurrence of certain pairs of residues, as compared to proteins and non-regulatory peptides.

Although these biochemical and bioinformatics approaches can provide useful information about neuropeptides and potentially identify new neuropeptides, if the cognate receptor is unknown, they will not be able to provide a general format for the computational identification of this class of hormones. Such a general format can be achieved using more sophisticated computational tools such as Hidden Markov Models.

Hidden Markov models were originally developed for speech recognition [30] and have long come to form the basis of the state of the art in that field. Estimation and hypothesis testing algorithms for HMMs have been well studied, and a wealth of experience makes it possible to train and test large-scale models from large amounts of data. Development of automatic speech recognition systems has motivated one of the key aspects of the presented approach in that there is a direct analogue between using hierarchical sentence, word, and phone hidden Markov models in speech recognition and the hierarchical modeling of functional elements in this work. A prohormone may be viewed as a sentence formed in a certain grammar using specific words, i.e. functional elements, which in turn are modeled by a sequence of phones, i.e. amino acids.

It is useful to differentiate between two usages of HMMs in biological sequence analysis: (1) *Pairwise-HMMs* [31] are a stochastic generalization of the sequence alignment algorithms and may be regarded as probabilistic model based counterparts of existing techniques, such as BLAST [32]. Their distinguishing characteristic is that as models they generate alignments of two sequences, their hidden states corresponding to insertions, deletions or substitutions. (2) *Profile HMMs* [33],[34] have proven to be a major breakthrough in biological sequence analysis, enabling modeling of protein families with a high degree of functional accuracy. For over a decade they have formed the basis of the most widely used applications of sequence modeling in molecular biology [35]. Profile HMMs, as models, generate a single sequence with a set of hidden states corresponding to the genomic structure of the molecule.

The basic computational units in this work are Match-profile HMMs (MPHMMs) [12],[36], which combine the capabilities of the two types of HMMs in that they can be viewed as using a profile HMM structure in modeling the sequence structure and a pairwise HMM (or a multiple-genome generalization thereof) in modeling the evolutionary characteristics of variation across species. In particular, the composite structure by which the preprohormone evolution is modeled is a *hierarchical grammar* of MPHMMs. Hierarchical grammars of MPHMMs are probabilistic models that take into account the manner in which substitutions take place in the evolutionary path at specific sites along the genome and the specific patterns of change from one site to the next. This kind of hierarchical alignment is significantly more informative than a conventional multiple sequence alignment (e.g., a la ClustalW) in that it provides segmentation of functional context. For example, for the peptide hormone problem, the most important feature of a cross-genome alignment turns out to be the difference between the substitution rates of the functional and the nonfunctional subsequences around (predominantly double basic residue) splicing sites.

There are several approaches in the literature for addressing similar problems. Phylogenetic HMMs, or phylo-HMMs, are probabilistic models that combine HMMs and phylogenetic trees in order to explain the spatial (genomic) and temporal (evolutionary) characteristics of a sequence, an excellent review of which is provided by Siepel and Haussler [13]. The first introduction of phylo-HMMs was motivated by the need to improve phylogenetic models that allow for variation in the substitution rate across sites [37],[38]; subsequently the problem of secondary structure prediction was addressed [39],[40]. Recently there is increased interest in these models as cross-genomic data become available in large quantities, and approaches that are informed by evolutionary pressures become enormously useful [41]–[45]. In particular, they have been applied to cross-genome gene prediction [46],[47]. Another similar structure is the evolutionary HMM [48],[49] that accounts for the phylogenetic information using generalizations of pairwise-HMMs, in a way similar to our approach. Evolutionary HMMs do not model the genomic structure in a targeted manner, as we do through the use of hierarchical grammars, and the spatial part of the model is used to track shifts in phylogenetic parameters.

Recently, a paper describing a different HMM-based method for the genomic identification of neuropeptides was published [50]. This paper used a single species method containing the peptide features we describe here, including a signal sequence, peptide, and prohormone cleavage site. The main difference of our approach from the published work is the use of cross-species comparisons through evolutionary models. In fact, there is prior work on discovery based on genomic structure alone. The problem of computational peptide hormone discovery based on genomic structure alone proves to be difficult. For example, an attempt to build models by specifying rules via deterministic grammars within the inductive logic grammar framework is described by Muggleton et al. [51]. In their manuscript, by enforcing the existence of signal sequences and splicing sites through a deterministic context-free grammar, a sieve for possible prohormone sequences is proposed. Even without the insight provided by evolutionary forces, the resulting method is able to eliminate unlikely candidates, but due to the ubiquitous existence of double basic residues throughout protein sequences, its selectivity turns out to be poor. In our approach, it is because of the signature of stochastic evolutionary pressures on the protein sequences that small functional peptide islands can be identified in the midst of a sea of diverged sequences.

In addition to our proof of principle using the Swiss Prot database, we have identified a number of potential preprohormones and their proposed processed neuropeptides. There were many unknown secreted proteins identified directly from the sequence matching protocol that fit the simple criterion of a pair of basic residues surrounding 4–50 amino acids. Visual examination of each possibility often detected reasons to decrease the likelihood that a particular protein was in fact a prohormone. There were three proteins for which we determined the presence of transcripts in the brain, and only one that was further characterized. Because preproNPQ, contains four potential biologically active agents, and because one of these was amidated, we considered this our most likely prohormone, with the most likely neurpeptide being the 14 amino acid amidated NPQ peptide (Figure 5). This peptide is conserved in mouse, dog, cow, and human sequences. It is conserved, except for a single amino acid change, as far back as fugu. The mRNA coding for this protein is found in brain, with higher levels in kidney.

Anatomical localization of preproNPQ mRNA with ISH demonstrated that its distribution is restricted to a very specific site in the brain (Figures 7 and 8). Our studies indicate that preproNPQ-containing cells overlap in their distribution with cells that express CRF in Barrington's nucleus, as well as those that are serotonergic and dopaminergic in the ventrolateral PAG. These results raise the possibility that preproNPQ may be co-localized in the same neurons with these neurotransmitters. Furthermore, this peptide is distributed closely to the cholinergic neurons of the mesopontine tegmentum raising the possibility that NPQ peptides may also interact with the cholinergic system.

The functional significance of these findings will require additional behavioral and physiological investigations, but it is reasonable to speculate that NPQ peptides may be involved in regulating a number of diverse functions. These likely include regulation of urinary, gastrointestinal and general autonomic functions, since Barrington's nucleus contains neurons that send polysynaptic projections to the bladder, colon, spleen and kidney [52],[53]. CRF-containing neurons in the Barrington's nucleus have been proposed to play a role in mediating stress-induced colonic alterations [54]. Based on the close overlap between CRF and preproNPQ, it seems feasible that NPQ peptides may play a role in the pathophysiology of stress-induced gastrointestinal disturbances.

Along the same lines of modulation of stress responses, dopaminergic (TH-positive) neurons of the ventrolateral PAG have been shown to project to the CRF-containing area of the bed nucleus of the stria terminalis (BNST) [55],[56], where these projections have been proposed to modulate CRF-initiated startle response [55]. Since we found close overlap between preproNPQ ISH signal and that for TH in the ventrolateral PAG, it is tempting to speculate that one of the NPQ peptides may play a role in regulating CRF-induced stress responses. The biological activity of the NPQ peptides is now under investigation.

Using a different HMM-based method for the genomic identification of neuropeptides, Mirabeau et al. identified two putative prohormones and processed peptides [50]. One peptide that was termed Spexin is identical to NPQ. They found spexin to co-localize with insulin in secretory granules, when transfected into rat pancreatic cells. ISH studies detected spexin mRNA only in the submucosal layer of the esophagus and stomach. Spexin mRNA was not reported in the brain. Finally, they showed that the amidated 14- amino acid peptide induced contractions of the rat fundus muscle of the stomach. This is an interesting observation, since our findings indicate that the rat almost certainly does not make amidated peptide because of the single amino acid change found within the C-terminal cleavage site (see Figure 5). Demonstration of functional activity of this compound in the rat stomach suggests that the C-terminal portion of NPQ is likely not involved in binding to its still unidentified receptor.

The computational method that led to the discovery Spexin identified another peptide that the authors named augurin [50]. Augurin is an uncharacteristically long peptide, 78 amino acids within a prohormone of length 148 amino acids. In terms of scoring, we are heavily penalizing peptides that are long with respect to their flanking non-functional sequences, and in the viewer, we have a filter that eliminates altogether any hit with length greater than 50% of the whole protein length. An experiment that modified our scoring and filters to test whether our model also works for augurin, verified that augurin was indeed detected by HIGHER as an instantiation of the following structure
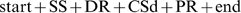
in our grammar.

There are other neuropeptides, which were not identified using our MP-HMM techniques. There are several potential reasons for other missing neuropeptides, the first and probably most important of which relates to the dataset used. The datasets of known and hypothetical proteins do not contain all the preprohormones. Although the genomes of mouse and human have been sequenced, the complement of predicted proteins is constantly changing and is different in the different databases. Another reason for not identifying prohormones is that the MP-HMM methodology utilized is statistical in nature and will not necessarily identify 100% of the target proteins. There are also many prohormones that do not have the classical profile of pairs of basic residues surrounding the neuropeptide. We are currently implementing a single basic residue algorithm based upon known splicing characteristics [18] that should lead to the identification of additional neuropeptides.

### Conclusion

We have presented a computational framework that is capable of accounting for protein structure and cross-species evolutionary divergence simultaneously. By aligning low-level evolutionary HMM modules within a high-level functional-element grammar, it is possible to build precise models of the effects of evolutionary pressures on genomic structures. In particular, we have applied this technique to modeling of prohormones across species with the goal of identifying novel prohormones and associated peptide hormones based on their evolutionary divergence profiles and genomic structures. This technique has resulted in high accuracy detection in a known dataset and led to putative hormones in a set of hypothetical proteins. Biochemical validation of the findings has resulted in the initial characterization of the prohormone preproNPQ, containing four potential previously undiscovered neuropeptides.

## Materials and Methods

### Polymerase Chain Reaction (PCR) of cDNA from Brain Using Species-Specific Primers

In order to determine if the putative transcript named preproneuropeptide Q (preproNPQ) is found in the brain, we performed PCR using rat, human and mouse specific primers with their corresponding cDNAs. The sequences of the primers used were: Rat Forward Primer 5′-GAAGGGGCCGAGCATCCTGG-3′ and Reverse Primer 5′-CACCAGTAAAAGCGTCTGTCTTC-3′; Mouse Forward Primer 5′-GGACAGGGTCGGAACATGAAG-3′ and Reverse Primer 5′-GTGTTTTCACCAGTTGAAGAGTC-3′; Human Forward Primer 5′-ACGCAGAACATGAAGGGACTCAGA-3′ and Reverse Primer 5′-CCAGTATATTTTCACCAGTTAAGC-3′. Advantage Genomic Polymerase Mix enzyme (BD Biosciences Clontech, CA) was used for PCR, according to manufacturer's instructions. Approximately 200–300 ng cDNA was used for each 50 ml reaction, along with 10 mM of specific forward and reverse primer, 2.2 ml magnesium acetate and dNTPs (10 mM). The annealing temperature was set at 53°C, and after 25 cycles of amplification, the PCR products were run on a 1.5% agarose gel and visualized using ethidium bromide. A positive control PCR reaction was also performed at the same time, using rat brain cDNA and specific primers for the prepronociceptin gene, and the reaction product was run on the gel.

### *In Situ* Hybridization in Rat Brain Slices Using Rat NPQ Probe

#### Tissue collection

Rats (n = 4) were killed via rapid decapitation using a guillotine. The brains were extracted, flash frozen in 2-methylbutane at −30°C, and stored at −80°C. Brains from each animal were cryostat sectioned coronally to a thickness of 10 µm at −20°C and thaw-mounted onto Superfrost slides (Fischer Scientific, Pittsburgh, PA). Slides were collected in sets of 10, and adjacent sections were placed on consecutive slides. This strategy allowed us to perform in situ hybridization (ISH) for different mRNAs on adjacent sections. The radioactive signal from these adjacent sections was digitally overlaid to determine regional localization of preproNPQ mRNA.

#### In situ hybridization (ISH)

Slides were removed from −80°C and placed in 4% paraformaldehyde at room temperature for 1 hour. Slides were washed 3 times in 2× SSC (300 mM NaCl/30 mM sodium citrate, pH 7.2) for 5 min, washed in 0.1 M TEA with 0.25% (vol/vol) acetic anhydride (pH 8.0) for 10 min, dehydrated through a series of alcohol washes (50%, 75%, 90%, 95% ×2, 100% ×2 EtOH, for 30 seconds each), and air dried. Radioactive probes for preproNPQ, tyrosine hydroxylase (TH; synthetic enzyme for dopamine and norepinephrine), tryptophan hydroxylase 2 (TPH2; synthetic enzyme for serotonin), choline acetyltransferase (ChAT; synthetic enzyme for acetylcholine), and corticotropin-releasing factor (CRF) were prepared from E. coli containing pBluescript SK cloning vectors (Stratagene, San Diego, CA), which were grown at 37°C for 16 hours in a shaker. The preproNPQ probe was designed to be 340 nucleotides in length. Those for TH, TPH2, ChAT and CRF were 274, 1030, 520, 762 nucleotides in length, respectively, and were based on publicly-available sequences downloaded from NCBI Entrez Gene (http://www.ncbi.nlm.nih.gov/entrez/query.fcgi?CMD=search&DB=gene).

To verify that the inserts were of predicted lengths, DNA was extracted and the inserts were excised with appropriate restriction enzymes. The products were separated by gel electrophoresis on a 2% agarose gel and visualized with ethidium bromide. Probes were also sequenced using dideoxynucleotide sequencing at the University of Michigan's DNA Sequencing Core. Sequenced products showed perfect alignment with predicted sequences.

For radioactive cRNA probe synthesis, DNA was extracted and then linearized. The reaction mix for both sense and anti-sense RNA probes contained the following: 4 µl of ^35^S-UTP (10 µCi/µl; Amersham Biosciences, Piscataway, NJ), 3 µl ^35^S-CTP (10 µCi/µl; Amersham Biosciences), 2.0 µl 5× transcription buffer, 1.0 µl 0.1 M DTT, 1.0 µl each of 10 mM ATP and GTP, 2.0 µl linearized plasmid DNA, 0.5 µl RNase inhibitor, and 1.5 µl T3 RNA polymerase, in a total reaction volume of 25 µl. The mixture was incubated at 37°C for 2 hours. After this period, 1 µl of RNase-free DNase was added to the mixture and allowed to incubate for an additional 15 min at room temperature. Each probe was then purified using column-based chromatography (BioRad Micro Bio-Spin Chromatography column, BioRad, Hercules, CA), and its radioactivity was quantified using a liquid scintillation analyzer. Following its preparation, each probe was diluted in hybridization buffer (50% formamide, 20% filtered water, 15% 20× SSC, 2% 50× Denhardt's solution, 2% tRNA, 10% 0.5 M sodium phosphate buffer, 10% dextran sulfate) and applied to dehydrated slides. A cover slip with 50–70 µl of hybridization buffer, 1–2×10^6^ DPM of radioactive probe, and DTT at a final concentration of 10 mM, was placed on each slide. Hybridization trays were prepared by lining the bottom of each tray with filter paper, which was saturated with 50% formamide buffer, and the slides were placed within. All trays were sealed and placed at 55°C overnight. Approximately 18 hours later, cover slips were removed and the slides were washed three times in 2× SSC for 5 min each. Next, slides were incubated in RNase A (200 µg/ml in 10 mM Tris-HCl, pH 8.0/0.5 M NaCl) at 37°C for 1 hour, then washed in a series of salt washes with increasing stringency: 2× SSC, 1× SSC and 0.5× SSC at room temperature for 5 min each, followed by a one-hour incubation in 0.1× SSC at 65–70°C. Finally, slides were dipped in distilled water and dehydrated through graded ethanol solutions: 30 seconds each in 50%, 75%, 90%, 95% ×2, and 100% ×2.

To determine the distribution of radioactive cRNA *in situ,* slides were apposed to radiosensitive film (Kodak Biomax; Eastman Kodak, Rochester, NY). Slides and the film were sealed within the cassette and stored in complete darkness. Following a 5–19-day exposure (exposure time depended on abundance of each mRNA species), films were developed using a Kodak X-OMAT 2000A processor (Eastman Kodak).

#### Image processing

ISH autoradiograms were digitized using a flatbed scanner (Microtek ScanMaker 1000XL, Microtek, Carson, CA) at 1600 dpi. Digital images were then inverted and each ISH signal was assigned a color as follows: TH – cyan, TPH2 – blue, preproNPQ – green, CRF – red, and ChAT – white. To determine regional co-localization of preproNPQ with the other mRNAs, images were then overlaid and aligned in Adobe Photoshop CS2 (Adobe Systems, San Jose, CA). Regional co-localization of signals was determined by mixing of the assigned colors. Illustrations were prepared in Photoshop and Adobe Illustrator CS2 (Adobe Systems). The signal was sharpened and brightness and contrast were adjusted for presentation purposes.

### Northern Blotting Using Human RNA Blot Probed with Human NPQ cDNA

In order to determine if the preproNPQ transcript could be detected in various human tissues, we used Ambion's First Choice Human Blot (a nylon membrane bound with 3 mg RNA from various human tissues, Ambion Inc, TX). The blot was prehybridized and probed with human NPQ cDNA prepared using the above preproNPQ human primers and the human DNA clone in pOTB7 vector from ATCC (Cat # 6710068, Manassas, VA). This clone contained the putative sequence for human preproNPQ, and the primers were used to isolate a 370 bp preproNPQ sequence that was used as the cDNA probe for hybridization to the RNA. Random-prime labeling of approximately 20–30 ng DNA was performed using ^32^P-dCTP and Klenow DNA polymerase, and after purifying the labeled probe on a G-50 column, the labeled DNA probe was hybridized to the nylon membrane overnight at 42°C. The membrane was washed and exposed to film.
